# Nervous system guides behavioral immunity in *Caenorhabditis elegans*

**DOI:** 10.7717/peerj.18289

**Published:** 2024-10-15

**Authors:** Yu Wang, Xuehong Sun, Lixiang Feng, Kui Zhang, Wenxing Yang

**Affiliations:** 1 Department of Physiology/West China School of Basic Medical Sciences & Forensic Medicine, Sichuan University, Chengdu, Sichuan, China; 2 Department of Forensic Pathology/West China School of Basic Medical Sciences & Forensic Medicine, Sichuan University, Chengdu, Sichuan, China

**Keywords:** *C. elegans*, Pathogen, Avoidance, Behavioral immunity

## Abstract

*Caenorhabditis elegans* is a versatile model organism for exploring complex biological systems. Microbes and the external environment can affect the nervous system and drive behavioral changes in *C. elegans*. For better survival, *C. elegans* may develop behavioral immunity to avoid potential environmental pathogens. However, the molecular and cellular mechanisms underlying this avoidance behavior are not fully understood. The dissection of sensorimotor circuits in behavioral immunity may promote advancements in research on the neuronal connectome in uncovering neuronal regulators of behavioral immunity. In this review, we discuss how the nervous system coordinates behavioral immunity by translating various pathogen-derived cues and physiological damage to motor output in response to pathogenic threats in *C. elegans*. This understanding may provide insights into the fundamental principles of immune strategies that can be applied across species and potentially contribute to the development of novel therapies for immune-related diseases.

## Introduction

In many model organisms, understanding the mechanisms of neuroimmune regulation is challenging due to the complexity of their nervous and immune systems, and the nervous system’s role in immune regulation is often obscured by adaptive immunity. *Caenorhabditis elegans* stands out as an ideal model organism for neuroimmune studies because of its simple, well-characterized nervous and immune systems. Its transparent body permits *in vivo* observation of cellular processes, and its genetic tractability allows for precise gene manipulation involved in neuroimmune interactions. Additionally, its short lifespan, ease of cultivation, and conserved signaling pathways with humans make it a valuable and cost-effective research model. *C. elegans* is a simple host organism that feeds on bacteria (Barrière & Félix, 2014; Samuel et al., 2016). The species composition of its bacterial food significantly influences various behaviors, such as feeding, locomotion, and chemotaxis (Chang et al., 2006; Sawin, Ranganathan & Horvitz, 2000). Therefore, the ability to distinguish between pathogenic and non-pathogenic microbes is essential for the survival and homeostasis of *C. elegans*.

Interestingly, naive *C. elegans* initially exhibit an innate attraction towards the odors of *Pseudomonas aeruginosa* PA14 (Ha et al., 2010; Zhang, Lu & Bargmann, 2005), *S. marcescens* (Pradel et al., 2007), *M. nematophilum* (Filipowicz, Lalsiamthara & Aballay, 2021), *E. faecalis* (Filipowicz, Lalsiamthara & Aballay, 2021), *S. aureus* (Irazoqui et al., 2010), *etc*., and then gradually learn to escape from them. This adaptive behavioral response, as a general strategy for *C. elegans* to respond to different pathogens, is known as behavioral immunity (Singh & Aballay, 2020), which refers to avoidance behaviors that protect against pathogen infections (Schaller, 2016). Behavioral immunity in *C. elegans* involves both innate pathogen recognition and internal state modulation due to intestinal infection (Meisel & Kim, 2014). If behavioral immunity doesn’t work, *C. elegans* may die from severe pathogen infection. As an illustration, PA14 may kill *C. elegans* in various ways, including host colonization, iron-induced hypoxic response, and damage to host translation (Feinbaum et al., 2012; Kirienko et al., 2013; Vasquez-Rifo, Ricci & Ambros, 2020).

Studying behavioral immunity in *C. elegans* is significant not only for understanding this specific organism but also for gaining insights into more complex systems. By elucidating the mechanisms of pathogen avoidance in this simple model, we can uncover fundamental principles of immune strategies that may be conserved across species. This knowledge could potentially contribute to our understanding of neuroimmune interactions in higher organisms and even inform the development of novel therapies for immune-related diseases in humans.

In this review, we discuss the role of the nervous system in behavioral immunity in *C. elegans*. We will explore how the nervous system translates the recognition of pathogen-derived cues to physical avoidance behavior, and focus on the integration of various signaling pathways and neural circuits. We aim to provide insights for understanding the complex interplay between the nervous system and immune responses, shedding light on the broader implications of behavioral immunity across different biological systems.

## Survey methodology

We searched the PubMed and Web of Science databases for all articles published before March 2024 on the association between behavioral immunity against pathogen infection and nervous system in *C. elegans*. The following terms were used in this search: ((elegans) AND (pathogen)) AND (neuron). To identify additional relevant publications, we also scanned the references cited in the research articles. Studies were included based on the following criteria: (1) investigation of the avoidance behavior of *C. elegans* towards one or more pathogens, and (2) identification of genes, signaling molecules, or neurons in the nervous system involved in the avoidance behavior in *C. elegans*.

## Neural mechanisms underlying behavioral immunity

**Figure 1:**
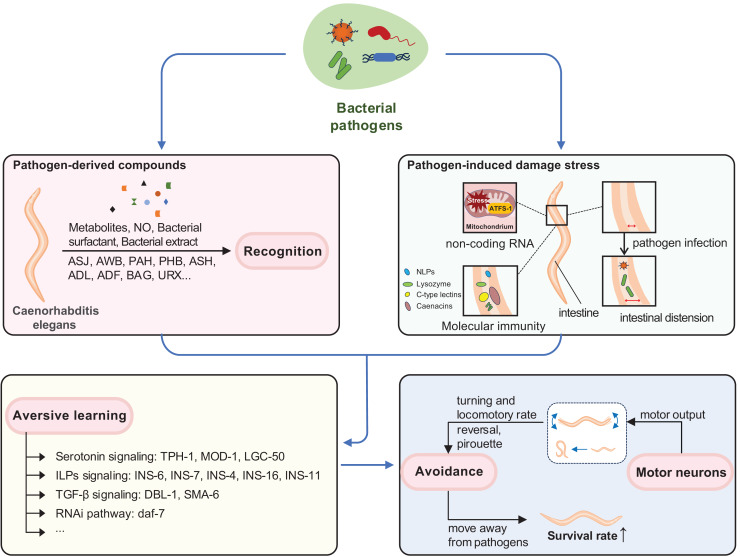
Neuroimmune response induced by pathogens in *C. elegans*. Behavioral immunity in *C. elegans* involves three interrelated processes: recognition of pathogen-derived cues, aversive olfactory learning, and generation of motor output. *C. elegans* can recognize pathogen-derived compounds (such as pathogen metabolites, NO, bacterial surfactant, bacterial extract) in the environment through sensory neurons. Infrequent exposure to pathogenic bacteria can trigger various stress responses and damage in *C. elegans*, such as mitochondrial stress and intestinal distension. Aversive learning enables *C. elegans* to link these pathogen-derived compounds with experiences of pathogenic injury through distinct pathways. When they encounter the same pathogen again, behavioral immunity is activated, promoting the motor output necessary for pathogen avoidance and enhancing survival.

Behavioral immunity in *C. elegans* involves three interconnected processes: recognition of pathogen-derived cues, aversive olfactory learning, and generation of motor output (Meisel & Kim, 2014). The signaling among sensory, inter-, and motor neurons is required to trigger behavioral immunity (Bretscher et al., 2011; Ha et al., 2010; Meisel & Kim, 2014; Samuel et al., 2016). In this section, we will provide an overview of how neural signals are processed, from pathogen-derived cue recognition to learning and motor output, before delving into the specific pathways involved (Fig. 1).

### Recognition of pathogen-derived cues

The sensory neurons and related neural circuits in *C. elegans* play a major role in sensing and integrating signals of food and potential danger, such as pathogens in the external environment. This integration is crucial for triggering an adequate behavioral output-whether to stay or leave-ensuring better survival (Fig. 1; Liu & Sun, 2021; Singh & Aballay, 2019a).

*C. elegans* identifies pathogenic bacteria by sensing various cues. While traditionally these cues might be referred to as pathogen-associated molecular patterns (PAMPs) in other systems, it’s important to note that *C. elegans* immunity doesn’t involve what we traditionally consider as PAMPs recognized by Toll-like receptors. In this review, we use the term “pathogen-derived compounds”, instead of PAMPs, to describe any compounds emitted by pathogens that *C. elegans* can detect and avoid. Additionally, *C. elegans* can also sense pathogen infection through the recognition of pathogen-induced cellular damage or stress (Matzinger, 2002; Pujol et al., 2008; Stuart, Paquette & Boyer, 2013).

#### Recognition of pathogen-derived compounds

To elicit behavioral immunity, *C. elegans* must recognize the encountered bacteria as a pathogen. This recognition process initially relies on sensing pathogen-generated compounds through its nervous system. According to previous studies, several bacterial species such as PA14 (Ha et al., 2010; Zhang, Lu & Bargmann, 2005), *S. marcescens* (Pradel et al., 2007), *B. thuringiensis* (Peng et al., 2018), *M. nematophilum* (Filipowicz, Lalsiamthara & Aballay, 2021), *E. faecalis* (Filipowicz, Lalsiamthara & Aballay, 2021), *S. aureus* (Irazoqui et al., 2010), *Streptomyces* (Tran et al., 2017), and *Oomycetes* (Fasseas et al., 2021) have been reported as *C. elegans* pathogens. Some of pathogen-derived compounds (Fasseas et al., 2021; Hao et al., 2018; Park, Meisel & Kim, 2020; Pradel et al., 2007; Prakash et al., 2021; Tran et al., 2017) have been identified (Table 1). These compounds can be categorized into two classes: non-gaseous organic compounds (*e.g*., phenazine-1-carboxamide, pyochelin, 1-undecene, Serrawettin W2, dodecanoic acid) and the gaseous inorganic compound nitric oxide (NO, Table 1). To sense non-gaseous organic compounds, *C. elegans* employs chemosensory neurons such as AWB, ASJ, ASH, ADL, ADF, ASK, PHA, and PHB. Interestingly, most of these neurons are known for sensing aversive stimuli (Liu et al., 2023; Tanimoto et al., 2017; Zou et al., 2017). For sensing the gaseous inorganic compound NO, ASJ sensory neurons are utilized (Hao et al., 2018). These findings highlight the importance of ASJ neurons, as they are currently the only pair of neurons known to respond to both types of pathogen-derived compounds.

**Table 1:** Pathogen-derived compounds.

Pathogen	Compound	Neuron involved	Molecule involved	Reference
PA14	Phenazine-1-carboxamide pyochelin	ASJ	DAF-7	Park, Meisel & Kim (2020)
PA14	1-undecene	AWB	/	Prakash et al. (2021)
PA14	NO	ASJ	GCY-27	Hao et al. (2018)
			DAF-11	
			TRX-1	
*S. marcescens*	Serrawettin W2	AWB	/	Pradel et al. (2007)
*Streptomyces*	Dodecanoic acid	ASH	SRB-6	Tran et al. (2017)
		ADL		
		ADF		
		PHA		
		PHB		
*oomycetes*	Innocuous extract	ASK	/	Fasseas et al. (2021)

Different neurons utilize different molecules to process signals from pathogen-derived compounds. For example, phenazine-1-carboxamide can induce rapid transcription of the *daf-7* gene, encoding a TGF-β ligand, in ASJ neurons through a G-protein-dependent signaling pathway (Park, Meisel & Kim, 2020). Increased levels of DAF-7 may act in conjunction with infection-associated modulation of internal state to facilitate subsequent avoidance behavior of *P. aeruginosa* and aid in reducing bacterial load by regulating oxygen sensing-behavior in *C. elegans* (Meisel et al., 2014; Singh & Aballay, 2019a). Additionally, ASJ neurons can sense PA14-derived NO through two kinds of receptor-type guanylate cyclase, DAF-11 and GCY-27 (Hao et al., 2018). These results suggest that neurons can respond to pathogen-derived compounds through either pre-existing molecules or newly induced molecules. Unfortunately, our knowledge of the receptors for these compounds is limited. Tran et al. (2017) identified SRB-6 as a potential receptor for the *streptomyces*-derived dodecanoic acid. Our previous work showed that DAF-11 and GCY-27 may be involved in NO-sensing in ASJ neurons, although we did not have direct data to support them as receptors for NO (Hao et al., 2018). Thus, it remains largely elusive how the *C. elegans* nervous system detects pathogen-derived compounds.

#### Sensing pathogen-induced cellular damage or stress

Apart from releasing harmful compounds, pathogen infection can cause cellular damage or stress, including mitochondrial stress and disruptions in protein biosynthesis, which *C. elegans* can detect. This process, sometimes referred to as “surveillance immunity”, involves monitoring core cellular processes for disruptions (Pukkila-Worley, 2016). It represents a sophisticated mechanism for *C. elegans* to identify pathogen invasion by sensing disturbances in essential cellular functions. The major regulators of stress responses in *C. elegans*, such as the p38 MAPK, DAF-16/FOXO, SKN-1/Nrf2, and HSF-1, play crucial roles in recognizing pathogen-induced damage-associated molecular patterns (DAMPs) and mounting immune responses against pathogens (Hajdú, Szathmári & Sőti, 2024; Yang et al., 2022a).

*C. elegans* intestinal epithelial cells function as a major pathogen recognition site for cellular damage or stress (Fig. 1). Some pathogens, such as *P. aeruginosa*, can disrupt host protein biosynthesis. For example, exotoxin A from *P. aeruginosa* can enter *C. elegans* intestinal epithelial cells and inhibit protein translation (Dunbar et al., 2012; McEwan, Kirienko & Ausubel, 2012). This inhibition leads to an increase in the level of the ZIP-2 bZIP transcription factor, which may regulate *C. elegans* immune response genes, including *irg-1* (Dunbar et al., 2012), *pmk-1* (Taouktsi et al., 2023), and *skn-1* (Grover et al., 2024).

**Figure 2:**
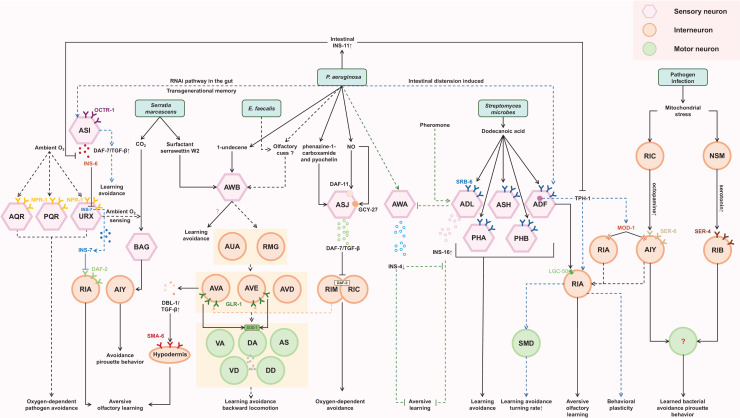
Cellular and molecular mechanisms of behavioral immunity. The sensory neurons, including ASJ, AWB, PAH, PHB, ASH, ADL, ADF, BAG, and URX, are responsible for recognizing pathogen-derived compounds. This sensory information is then transmitted to downstream interneurons such as RIA, RIY, RIM, RIC, AVA, AVE, AVD, AIZ, AIY, RIB, NSM, and RMG. Ultimately, motor output is directed by motor neurons like SMD, VA, DA, AS, VD, and DD. Throughout this process, various molecular pathways, *e.g*., TGF-β signaling and insulin-like peptides, play critical roles.

Mitochondria, central organelles for energy production and metabolism, are common targets of pathogen attack in intestinal epithelial cells (Fig. 1) (Chen et al., 2021; Liao et al., 2022). Exposure to pathogens like PA14 can cause mitochondrial dysfunction and activate the mitochondrial unfolded protein response, UPR(mt) (Chen et al., 2021; Liao et al., 2022; Pellegrino et al., 2014). *C. elegans* monitors the import efficiency of the transcription factor ATFS-1, which mediates UPR(mt), to sense potential pathogen infection. Inhibition of mitochondrial functions induces bacterial avoidance (Liao et al., 2022; Liu et al., 2014; Pellegrino et al., 2014). During mitochondrial stress, ATFS-1 induces not only mitochondrial protective genes to mediate UPR(mt) but also innate immune genes, including a secreted lysozyme and antimicrobial peptides, as well as two C-type lectins involved in pathogen recognition (Fig. 2) (Pellegrino et al., 2014).

### Aversive olfactory learning to pathogens

Aversive olfactory learning plays a vital role in the behavioral immunity in *C. elegans*. This process allows the nematode to associate physiological responses, such as intestinal distension and immune activation, with pathogen-derived cues (Filipowicz, Lalsiamthara & Aballay, 2021; Filipowicz, Lalsiamthara & Aballay, 2022; Kaletsky et al., 2020; Reddy et al., 2009; Singh & Aballay, 2019a). Recent studies have revealed complex cellular and molecular pathways involved in this learning process.

#### Cellular mechanism underlying pathogen-mediated aversive olfactory learning

When *P. aeruginosa* or *E. faecalis* infects the intestine of *C. elegans*, it triggers the development of a learned aversive response (backward locomotion). This reflexive behavior requires the involvement of a four-layer aversive learning circuit in the nematode (Filipowicz, Lalsiamthara & Aballay, 2022). The olfactory signal from *P. aeruginosa* and *E. faecalis* is detected by AWB neurons and transmitted sequentially through electrical synapses to second-layer AUA and RMG neurons, chemical synapses to third-layer AVA, AVD, and AVE command interneurons, and finally to fourth-layer motor neurons, namely the VA, DA, VD, DD, and AS neurons, which are crucial for backward locomotion (Filipowicz, Lalsiamthara & Aballay, 2022). These motor neurons ultimately facilitate the backward movement necessary for avoiding the detected odor. Additionally, aversive learning to *P. aeruginosa* requires AWC, ADF, AIY, AIB, RIA neurons (Ha et al., 2010), while aversive learning to *E. faecalis* requires ASE and AWC neurons (Filipowicz, Lalsiamthara & Aballay, 2021).

Despite the intricate and bacteria-dependent nature of the neurons involved, both *P. aeruginosa* and *E. faecalis* engage the AWB neurons, which can respond to both attractive and aversive olfactory cues (Yang et al., 2022b), as well as the AWC neurons, which generally respond to attractive olfactory stimuli, for initial olfactory recognition. The signal transmission from these pathogens appears to activate a series of neurons at each layer of signal propagation, suggesting that the circuit likely intersects in a complex manner. This intricate interconnectivity makes it challenging to dissect the complete aversive learning circuit specific to a particular pathogen.

#### Molecular mechanism underlying pathogen-mediated aversive olfactory learning

**1) Biogenic amine signaling.**

The tyramine produced by the intestinal bacteria *Providencia* can be converted to octopamine, which then acts on the octopamine receptor OCTR-1 present in the ASH neurons (O’Donnell et al., 2020). The ASH neurons are a pair of neurons primarily known for sensing aversive stimuli. This interaction between octopamine and the ASH neurons leads to altered food choices in the nematode.

Serotonin signaling plays a crucial role in aversive olfactory learning to PA14 and *S. marcescens*. Prolonged exposure to either of them upregulates TPH-1, a serotonin biosynthetic enzyme, in ADF neurons. Serotonin secreted from ADF acts through MOD-1, a serotonin-gated chloride channel expressed in interneurons AIY/AIZ, to promote aversive learning (Zhang, Lu & Bargmann, 2005). When exposed to PA14, a redistribution of LGC-50, a serotonin-gated cation channel, occurs within RIA neuronal processes. This redistribution is crucial for the aversive olfactory learning associated with PA14 (Morud et al., 2021). These findings suggest that the intracellular trafficking and synaptic localization of LGC-50 may represent a molecular cornerstone underlying the learning mechanism. Furthermore, RIA and AIZ neurons, acting as postsynaptic targets of the serotonergic ADF neurons, express serotonin-gated chloride and cation channels, respectively. This suggests that when pathogen stimulates the ADF neurons to release serotonin, it may simultaneously suppress RIA while activating AIZ. This provides a model for how serotonin finely modulates the downstream neuronal state.

**2) Insulin and insulin-like peptide (ILP) signaling**.

ILP pathway plays significant roles in regulating aversive olfactory learning, developmental processes, lifespan, reproduction, and neural plasticity across various species (Chen et al., 2013; Cornils et al., 2011; Hwangbo et al., 2004). INS-6 and INS-7, members of the type-beta class of the *C. elegans* ILP superfamily, contribute antagonistically to aversive olfactory learning to PA14 through an ILP-to-ILP signaling pattern (Chen et al., 2013). INS-6, produced by the ASI sensory neurons, can suppress the transcription of *ins-7* in the URX neurons, thereby facilitating olfactory learning (Chen et al., 2013; Liu et al., 2018). High levels of INS-7, secreted by the URX neurons, disrupt olfactory learning by counteracting the function of DAF-2 in the postsynaptic RIA neurons (Chen et al., 2013). Additionally, INS-4 and INS-16 provide balanced signaling from the bacteria-sensing neuron AWA and the pheromone-sensing neuron ADL, respectively. This balanced signaling is critical for aversive olfactory learning of PA14 (Wu et al., 2019). These findings suggest that ILPs may interfere with each other, allowing for fine-tuning of neuronal functions. Furthermore, exposure to PA14 induces the expression of INS-11 in the intestine, which may contribute to aversive learning behavior by inhibiting the expression of TPH-1 in the ADF neurons and INS-6 in the ASI neurons (Lee & Mylonakis, 2017). This implies that the ILP pathway can interact with the serotonergic pathway as well.

**3) TGF-β signaling.**

DBL-1, a well-characterized *C. elegans* TGF-β ligand, has been implicated in aversive olfactory learning to PA14. Elevated expression of DBL-1 in AVA command interneurons may facilitate aversive olfactory learning through the type I TGF-β receptor SMA-6 in the adult hypodermis (Zhang & Zhang, 2012).

**4) RNAi-mediated learning**.

An intriguing mechanism of aversive learning involves the ingestion of small RNAs (sRNAs) from pathogenic bacteria by the *C. elegans* gut. These sRNAs operate through the RNA interference (RNAi) pathway in the gut, leading to signaling that involves the piRNA pathway and epigenetic modifiers. This signaling upregulates the expression of *daf-7* in the ASI neurons, ultimately inducing avoidance of PA14 in a manner independent of innate immunity. Remarkably, this sRNA-mediated memory can be transmitted across generations (Fig. 2) (Kaletsky et al., 2020).

### Motor output for pathogen avoidance

The final step in behavioral immunity is the generation of appropriate motor output that allow *C. elegans* to avoid pathogens. A key factor in triggering this avoidance behavior is the bloating of the intestinal lumen, induced by bacterial colonization and gut virulence factors (Singh & Aballay, 2019b). Animals detect intestinal lumen bloating as a danger signal, which then triggers various defense responses, such as activating stress response pathways, inducing behavioral changes, and upregulating antimicrobial peptides (Dierking et al., 2011; Hajdú et al., 2021; Kumar et al., 2019; Singh & Aballay, 2019b). The bloating of the intestinal lumen during infection drives various motor outputs such as reversal, pirouette behavior, and changes in locomotory or turning rate (Singh & Aballay, 2019a). These behavioral changes promote movement away from pathogens. Our understanding of which motor neurons connect the upstream signals to the learned pathogen avoidance behavior and how they do so is limited, but SMD, DA, and DB motor neurons are candidate motor neurons guiding the pathogen avoidance behaviors to PA14 or *M. nematophilum* (Fig. 2) (Ha et al., 2010; McMullan, Anderson & Nurrish, 2012; Yu & Chang, 2022). The intestinal bloating could induce multiple neuroendocrine pathways associated with pathogen avoidance, including the DAF-7/TGF-β pathway, NPR-1/G protein-coupled receptor (GPCR) pathway and TPH-1 pathway, indicating the complexity of neuroendocrine signaling in coordinating behavioral immunity responses.

Specifically, the DAF-7/TGF-β pathway in ASJ neurons is required to induce the behavioral avoidance of *P. aeruginosa*, activated by GPCRs (Meisel et al., 2014). DAF-7 may bind to the TGF-β type I receptor DAF-1 and the TGF-β type II receptor DAF-4, which then antagonize the co-SMAD DAF-3 in the adjacent RIM/RIC interneurons (Estevez et al., 1993; Georgi, Albert & Riddle, 1990). Increasing *daf-7* levels may enhance the inhibition of *daf-3* in RIM/RIC interneurons, modifying the response of *C. elegans* to oxygen levels and promoting exit from the low-oxygen environment created by *P. aeruginosa* metabolism. Although the downstream signaling output of RIM/RIC interneurons is not well illustrated, RIM interneurons have been reported to regulate the activities of AVA/AVE command interneurons (Kato et al., 2015; Li et al., 2023; Piggott et al., 2011). Correlatively, a study of the aversive sensorimotor circuitry in *P. aeruginosa* and *E. faecalis* describes AVA and AVE interneurons as acting upstream of VA, DA, AS, VD and DD motoneurons to coordinate backward locomotion (Filipowicz, Lalsiamthara & Aballay, 2022), suggesting that intersecting neural circuits are required for behavioral immunity against different pathogens. The AMPA-type ionotropic glutamate receptor GLR-1, expressed in AVA and AVE command interneurons, may target the superoxide dismutase SOD-1 in DA and DB cholinergic motoneurons to promote avoidance behavior (Yu & Chang, 2022), indicating a key role of glutamate signaling in mediating behavioral immunity in *C. elegans*.

Additionally, the NPR-1/GPCR, related to mammalian neuropeptide Y receptors, regulates the behavioral response of *C. elegans* to oxygen. Expression of *npr-1* in the URX, AQR and PQR oxygen-sensing neurons can elicit oxygen-dependent pathogen avoidance (Chang et al., 2006; Cheung et al., 2005; Reddy et al., 2009; Reddy et al., 2011). The AUA and RMG neurons are synaptic targets of URX, suggesting that DA, AS, VD and DD motoneurons are probably also related to this neural circuit (Filipowicz, Lalsiamthara & Aballay, 2022). The FMRF-like peptides FLP-18 and FLP-21, ligands of NPR-1, may function redundantly in this behavior, although they activate NPR-1 in social feeding and behavioral quiescence, and FLP-18 may control the reversal length through the NPR-1 receptor in ASE sensory neurons (Fig. 2) (Bhardwaj et al., 2018; Choi et al., 2013; Rogers et al., 2003).

Furthermore, the serotonergic ADF neurons play an essential role in regulating aversive olfactory learning of pathogenic bacteria, and the RIA interneuron and SMD motor neurons act downstream of the ADF to increase the rate of turning and thus reduce pathogen ingestion, contributing to the development of behavioral immunity (Chang et al., 2006; Ha et al., 2010; Zhang, Lu & Bargmann, 2005). RIA interneurons are believed to regulate behavioral plasticity in temperature and chemical sensation (Mori & Ohshima, 1995; Stetak et al., 2009), and may integrate the sensory and motor signals in their axon domain (Liu et al., 2018). Under mitochondrial insult, serotonin signaling from NSM neurons *via* the SER-4 serotonin receptor in RIB interneurons and octopamine signaling from RIC interneurons through the SER-6 receptor in AIY may function in parallel to modulate pirouette (high-angle turn) as a critical locomotor strategy to promote learned bacterial avoidance, although the specific locomotor elements they regulate remain unknown (Chiang, Liao & Pan, 2022; Liao et al., 2022). These studies suggest that an enhanced innate immune response is a consequence of changing in physiological state, which may govern, not only manipulate, aversive memory and behavior. Notably, AIY acts downstream of RIC to integrate sensory information, and has also been reported to function downstream of AWA/B/C, ASEL and BAG neurons to mediate avoidance to repulsive odors, salty and CO_2_, respectively, by inducing a pirouette head movement (Chalasani et al., 2007; Gray, Hill & Bargmann, 2005; Kocabas et al., 2012; Liao et al., 2022; Rengarajan et al., 2019; Wang et al., 2017), suggesting that they may share common downstream interneurons and motor neurons (Fig. 2).

## Conclusion

The studies presented in this review demonstrate the intricate and extensive connections between the nervous system and behavioral immunity in *C. elegans*. They highlight the role of conserved neuronal circuits in detecting potential pathogens and subsequently triggering pathogen avoidance to reduce the risk of infection, thus ensuring the survival of *C. elegans* against a diverse range of pathogens.

Several key points emerge from this review: (1) The nervous system of *C. elegans* integrates various signals, including pathogen-derived cues and internal physiological states, to coordinate behavioral immunity. (2) Multiple overlapping neural circuits are involved in behavioral immunity, with individual neurons often serving different roles depending on the context and the specific pathogen encountered. (3) Various neuroendocrine pathways, including TGF-β, insulin-like peptides, and monoamine signaling, play crucial roles in modulating behavioral immunity. (4) Aversive olfactory learning is a key component of behavioral immunity, allowing *C. elegans* to adapt its responses based on past experiences with pathogens.

Future research directions in this field could include the following five aspects. First, while many components of the behavioral immunity circuits have been identified, there is still much to learn about how these circuits integrate information and generate specific behavioral outputs. Second, the molecular underpinnings of aversive olfactory learning, particularly how long-term memories are formed and maintained, warrant further investigation. Third, the role of epigenetic modifications in behavioral immunity, especially in the context of transgenerational inheritance of learned behaviors, is an exciting area for future research. Fourth, further exploration of how behavioral immunity interacts with other aspects of *C. elegans* physiology, such as metabolism, aging, and reproduction, could provide valuable insights. Finally, investigating how the principles of behavioral immunity in *C. elegans* apply to other organisms could help identify conserved mechanisms and evolutionary adaptations.

In conclusion, the study of behavioral immunity in *C. elegans* provides a powerful model for understanding the complex interactions between the nervous system and immune responses. The insights gained from this simple organism have broad implications for our understanding of neuroimmune interactions across species and may contribute to the development of novel therapeutic approaches for immune-related disorders in more complex organisms, including humans.

## Additional Information and Declarations

### Data Availability

The following information was supplied regarding data availability: This is a literature review.
